# A New Species of the *Cyrtodactylus pulchellus* Group (Squamata: Gekkonidae) from Surat Thani Province, Thailand Underscores This Group’s Remarkable Diversity on the Thai-Malay Peninsula †

**DOI:** 10.3390/ani14223226

**Published:** 2024-11-11

**Authors:** Korkhwan Termprayoon, Attapol Rujirawan, Larry Lee Grismer, Anchalee Aowphol

**Affiliations:** 1 School of Science, Walailak University, Nakhon Si Thammarat 80161, Thailand; termprayoon_kk@yahoo.com; 2 Animal Systematics and Ecology Speciality Research Unit, Department of Zoology, Faculty of Science, Kasetsart University, Bangkok 10900, Thailand; fsciapr@ku.ac.th; 3 Biodiversity Center, Kasetsart University, Bangkok 10900, Thailand; 4 Herpetology Laboratory, Department of Biology, La Sierra University, 4500 Riverwalk Parkway, Riverside, CA 92515, USA; lgrismer@lasierra.edu; 5 Department of Herpetology, San Diego Natural History Museum, P.O. Box 121390, San Diego, CA 92112, USA; 6 Institute for Tropical Biology and Conservation, Universiti Malaysia Sabah, Jalan UMS, Kota Kinabalu 88400, Malaysia

**Keywords:** bent-toed gecko, genetic, Southeast Asia, taxonomy

## Abstract

An integrative taxonomic study recognized a new species of the *Cyrtodactylus pulchellus* group from Kanchanadit District, Surat Thani Province, Southern Thailand. Phylogenetic analyses of the mitochondrial *ND2* gene revealed a new species, *Cyrtodactylus kanchanadit* **sp. nov.**, that is closely related to *C. lekaguli*. The new species differs from its sister lineage by bearing mean genetic divergence of 4.53% and significant differences in both mensural and morphometric characters (*p* < 0.05). *Cyrtodactylus kanchanadit* **sp. nov.** can be differentiated from the remaining members of the *C. pulchellus* species group because of its genetic divergence of ≥7.99% and a combination of differences in body size: 11–15 supralabial and 10–12 infralabial scales, 29–35 paravertebral tubercles, 18–23 longitudinal rows of dorsal tubercles, 32–37 rows of ventral scales, 20–23 subdigital lamellae on the fourth toe, 32–37 femoroprecloacal pores in adult males, and four or five dark body bands; weak tuberculation on body; absence of tubercles in the ventral body fold, absence of precloacal pores in females, and absence of scattered pattern of white tubercles on dorsum; presence of precloacal groove depression; and maculated white caudal bands. *Cyrtodactylus kanchanadit* **sp. nov.** is currently known from its type locality.

## Simple Summary

This study discovered a new *Cyrtodactylus* population from Phet Phanomwat Waterfall in Southern Thailand. It has genetic divergences pertaining to mitochondrial NADH dehydrogenase subunit 2 (*ND2*) gene of ≥3.97% from its sister lineage, *C. lekaguli*, and bearing ≥ 7.99% genetic divergence from the congeners in the *C. pulchellus* group. This population can be separated from *C. lekaguli* by its morphologically significant differences. Based on the molecular and morphological evidence, this population is described as a new species, *Cyrtodactylus kanchanadit* **sp. nov.** The discovery of this new species increases the total number of *Cyrtodactylus* species to 377, of which 57 occur in Thailand.

## 1. Introduction

The genus *Cyrtodactylus* has been recognized for its remarkable diversity due to its adaptive ability in various habitats (e.g., limestone karst, granitic forest, swamps, vegetation, and lowland evergreen forest) and highly diverse morphology [1,2,3]. Over the decades, extensive exploration of previously unsurveyed areas revealed a vast number of unknown species that have been later described [4,5,6,7]. The discoveries continuously increase the members of this genus to 376 named species [8,9,10,11,12] and makes this genus one of the most diverse genus-level groups of vertebrates [2]. Due to its high adaptability, this genus is broadly distributed from South Asia to Melanesia, covering at least eight geographic regions [1,3,13]. Southeast Asia (including Malaysia, Laos, Myanmar, Cambodia, and Thailand) is recognized as a major global biodiversity hotspot [14] and home to over 180 *Cyrtodactylus* species within 21 of 31 species groups [2,8].

One of the Southeast Asian species groups is the *C. pulchellus* group occurring throughout the Thai–Malay Peninsular region. This species group is a monophyletic group containing 19 nominal species [15,16,17,18,19,20,21]. Most members are recognized as karst- and granite-associated species (except *C. macrotuberculatus*) that independently evolved from a generalist ancestor [1,17]. Despite the growing number in this species group resulting from unexplored areas from lowlands to mountain regions (e.g., [22]), several poorly known areas have not been visited, which are hypothesized to contain hidden diversity along its range. Our recent field surveys discovered a population from Kanchanadit District, Surat Thani Province, Southern Thailand. The morphology of the specimens collected closely resembles those species within the *C. pulchellus* group in various aspects. Molecular analyses showed it to be a different lineage from *C. lekaguli* Grismer, Wood, Quah, Anuar, Muin, Sumontha, Ahmad, Bauer, Wangkulangkul, Grismer, and Pauwels, 2012, known from this area, and additional multivariate and univariate analyses showed statistically significant differences between this new population and closely related species. Based on the provided evidence, specimens of the Kanchanadit population are considered to be a new species and are described below.

## 2. Materials and Methods

### 2.1. Sampling

**Figure 1:**
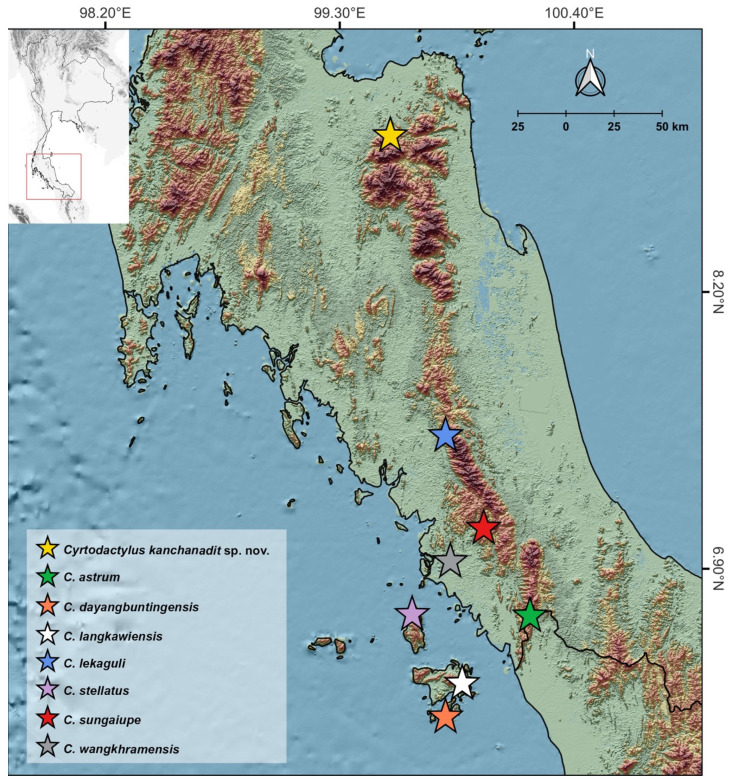
Map illustrating the sampling location of the newly discovered *Cyrtodactylus kanchanadit* **sp. nov.** (yellow star) in Kanchanadit District, Surat Thani Province, along with the type localities of closely related species within Clade A of the *C. pulchellus* group.

The visual encounter surveys were carried out at the Phet Phanomwat Waterfall in Surat Thani Province, Southern Thailand (Figure 1). Nine *Cyrtodactylus* specimens were collected between 1900 and 2100 h on 26 January 2019. Garmin GPSMAP 64s (Garmin Ltd., Olathe, KS, USA) was used to determine geographical coordinates and elevation. A Kestrel 400 Weather Meter (Nielsen-Kellerman Co., Boothwyn, PA, USA) was used to measure the ambient temperature and humidity, and ecological data were noted individually (i.e., substrate use, microhabitat, and time of capture). Specimens were photographed live and then euthanized using tricaine methanesulfonate (MS-222, Sigma-Aldrich Co., St. Louis, MO, USA). Tissue samples were removed and preserved in 95% ethanol for molecular study. Voucher specimens were fixed in 10% formalin, washed in running water, and then transferred to 70% ethanol for long-term storage. The vouchers were deposited in the herpetological collections of the Zoological Museum, Kasetsart University, Thailand (ZMKU).

### 2.2. Molecular Analyses

From the newly collected specimens, total genomic DNA was extracted from ethanol-preserved tissues using a NucleoSpin Tissue Kit (Macherey-Nagel GmbH & Co. KG, Dueren, Germany) according to the manufacturer’s standard protocols. The amplification was performed on the partial sequences of the mitochondrial *ND2* gene and flanking tRNAs using PCR condition following Termprayoon et al. [21] with a primer pair of MetF6 and COIH [23]. The products were purified using QIAquick^®^ PCR Purification Kit (Qiagen, Hilden, German), and the amplifying primers were used for sequencing in the ABI 3730XL DNA Sequencer by Sangon Biotech Inc. (Shanghai, China) using BigDye version 3 chemistry (Applied Biosystems, Carlsbad, CA, USA). GenBank accession numbers, museum numbers, and locality of specimens used are available in Table S1.

A total of 99 homologous sequences were downloaded from GenBank consisting of seven outgroup species and 92 selected individuals of other species in the *C. pulchellus* group.

The outgroup species (*Agamura persica* (Duméril, 1856), *Hemidactylus frenatus Duméril & Bibron*, 1836, *Tropiocolotes steudneri* (Peters, 1869), *C. elok* Dring, 1979, *C. hontreensis* Ngo, Grismer & Grismer, 2018, *C. interdigitalis* Ulber, 1993, and *C. intermedius* (Smith, 1917)) used to root the tree were selected by following Wood et al. [24]. The new and downloaded sequences were aligned using the MUSCLE plug-in implemented in Geneious Prime 2022.2.1. The amino acids of the partial *ND2* gene were translated to verify the sequences. All sequences used are listed in Table S1.

Maximum Likelihood (ML) and Bayesian Inference (BI) methods were performed to reconstruct phylogenetic trees. The best substitution models for each codon position and tRNAs were identified using ModelFinder with the Bayesian Information Criterion (BIC) embedded in IQ-TREE [25]. The selected models for ML were HKY + F + I + G4 for 1st, TIM3 + F + G4 for 2nd, and TN + F + G4 for 3rd coding position and K2P + G4 for the tRNAs. The ML tree was run in IQ-TREE webserver [26] with 1000 ultrafast bootstrap (UFB, [27]) replicates. The BI analysis was conducted using MrBayes 3.2.6 on XSEDE [28] through the CIPRES Science Gateway v.3.3 [29], employing the selected model that closely matched the models developed for the ML analysis. The Markov Chain Monte Carlo (MCMC) was run with four chains, three hot and one cold, for 10 million generations and sampled every 1000 generations. The first 25% of each tree was excluded as burn-in. Tracer v. 1.7.1. [30] was used to check the stationarity and the effective sample sizes (ESSs) for all parameters to be sure that all were above 200. With pairwise deletion, MEGA 11 [31] was used to compute uncorrected pairwise sequence divergences (*p*-distance), both intra- and interspecific.

### 2.3. Morphological Measurement

Morphological data were collected from the newly collected specimens. Sixteen morphological characters were measured on the left side of the body using Mitutoyo digital vernier calipers (to the nearest 0.1 mm, Mitutoyo Co., Kawasaki, Japan), and eighteen meristic characters were identified under a Nikon SMZ745 (Nikon Co., Tokyo, Japan) dissecting microscope on both left (L) and right (R) sides, when possible. Characters and their abbreviations follow Termprayoon et al. [21] (see Abbreviation).

Additional non-metric categorical characters that were considered were the degree of body tuberculation, and weak tuberculation refers to dorsal body tubercles that are low and rounded, whereas prominent tuberculation refers to tubercles that are raised and keeled; the presence or absence of tubercles on the dorsal and ventral surfaces of the forearms; the presence or absence of tubercles in the gular region, throat, and ventrolateral body folds; body tubercles extending past the base of the tail; the width of the dark body bands relative to the width of the interspace between the bands; the presence or absence of dark pigmentation infused in the white caudal bands of adults; the presence of caudal tubercles; the presence or absence of a precloacal depression or groove; femoroprecloacal pores that are continuous or discontinuous; the presence or absence of scattered white/yellow tubercles on the dorsum; and the presence or absence of white tail tip in hatchlings and juveniles. The color pattern was assessed for dorsal, ventral, and lateral images of the body in both sexes and for all possible age classes before preservation.

### 2.4. Statistical Analyses

Thirty preserved specimens of the Kanchanadit population (*n* = 6) and *C. lekaguli* (*n* = 24) were used for statistical analysis. R studio version 2023.12.1 was used for all statistical analyses [32]. Fifteen mensural characters were size-adjusted using an allometric equation: X_adj_ = log[X ± β(SVL ± SVL_mean_)], where X_adj_ = adjusted value; X = measured value; β = unstandardized regression coefficient for each species; SVL = measured snout–vent length; and SVL_mean_ = overall average SVL of each allometry species [33,34,35,36] implemented through the R package GroupStruct [37]. Tail length (TL) was rejected for analyses due to their condition (e.g., original, regenerated, or broken tail). The adjustment was conducted for each species and then concatenated into a single data frame to ensure there is no interspecific conflation of variation [38,39]. Prior to the analyses, all 15 adjusted characters and seven meristics (SL, IL, PVT, LRT, VS, 4TL, and BB) were merged into a single dataset. The other characters were omitted as data for some specimens were unavailable.

The dataset was analyzed by principal component analysis (PCA) using the factorMine R package [40] to reduce its complexity and to select a subset of input variables that retain the most significant information. The plots were visualized using *ggplot2* package [41]. The differences between the Kanchanadit population and *C. lekaguli* were compared using both parametric *t*-test and non-parametric Mann–Whitney U test. Dataset was checked for normal distribution using the Shapiro–Wilk test (*p* ≥ 0.05) and equality of variance using the Levene’s test (*p* ≥ 0.05). A comparison was performed by *t*-test for normalized and equal variance data or Welch’s *t*-test for unequal data. Non-parametric Mann–Whitney U test was conducted for non-normalized data. A non-parametric permutation-multivariate analysis of variance (PERMANOVA) from *vegan* package 2.5-3 [42] was used to determine the significant differences in centroid locations and group clustering between species based on the loadings of the first four dimensions recovered from the PCA. The analysis computes a Euclidean (dis)similarity matrix with 50,000 permutations.

## 3. Results

### 3.1. Phylogenetic Relationships

**Figure 2:**
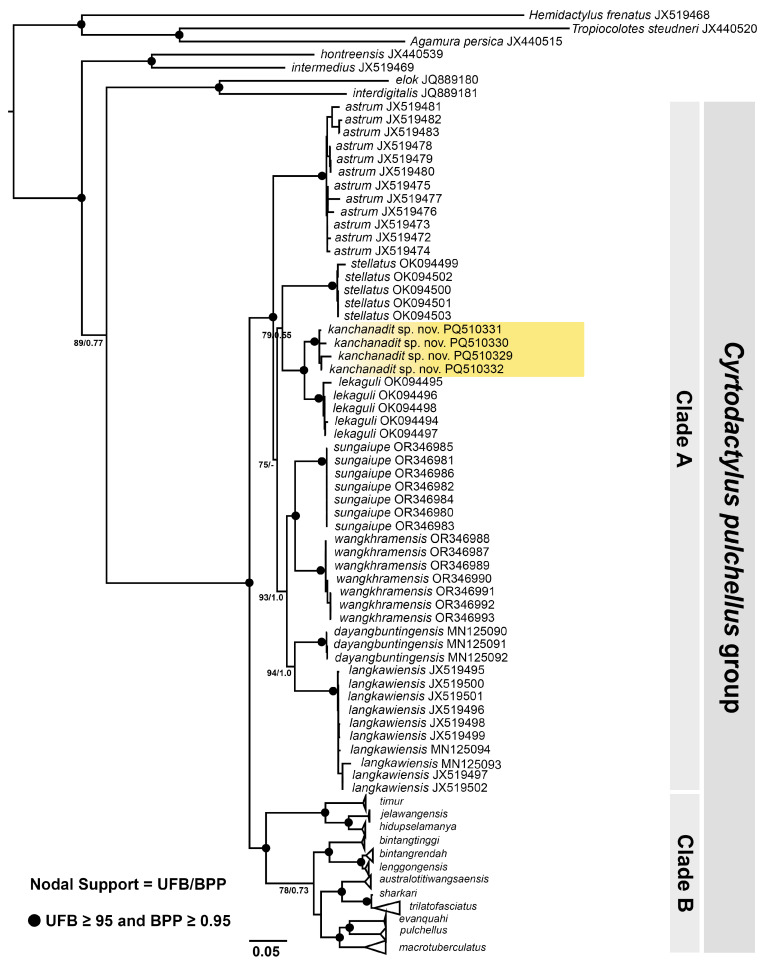
Maximum Likelihood phylogenetic tree of *Cyrtodactylus kanchanadit* **sp. nov.** and the other members of *C. pulchellus* group based on mitochondrial *ND2* and flanking tRNA of 99 specimens. Support values at nodes are ultrafast bootstrap (UFB) and Bayesian posterior probability (BPP).

The phylogenetic ML and BI analyses recovered largely identical topologies with slightly different support values. The ML tree with UFB and BPP is shown in Figure 2. Both analyses showed that the Kanchanadit population is a member of the *C. pulchellus* group and is deeply nested within Clade A consisting of *C. astrum* Grismer, Wood, Quah, Anuar, Muin, Sumontha, Ahmad, Bauer, Wangkulangkul, Grismer, and Pauwels, 2012, *C. dayangbuntingensis* Quah, Grimer, Wood, and Sah, 2019, *C. langkawiensis* Grismer, Wood, Quah, Anuar, Muin, Sumontha, Ahmad, Bauer, Wangkulangkul, Grismer, and Pauwels, 2012, *C. lekaguli*, *C. stellatus* Termprayoon, Rujirawan, Ampai, Wood, and Aowphol, 2021, *C. sungaiupe* Termprayoon, Rujirawan, Grismer, Wood, and Aowphol, 2023, and *C. wangkhramensis* Termprayoon, Rujirawan, Grismer, Wood, and Aowphol, 2023.

The Kanchanadit samples formed a strongly supported monophyletic lineage (100 UFB, 1.00 BPP) and were recovered as a sister lineage (100 UFB, 1.00 BPP) to *C. lekaguli*. The Kanchanadit population had *p*-distances of 3.97–5.55% from *C. lekaguli* and ≥ 7.99% from the remaining species in Clade A. The *p*-distances within the Kanchanadit population were 0.52 to 1.78% (mean: 1.23%). The *p*-distances between and within species in Clade A of the *C. pulchellus* group are shown in Table 1.

### 3.2. Morphology

**Figure 3:**
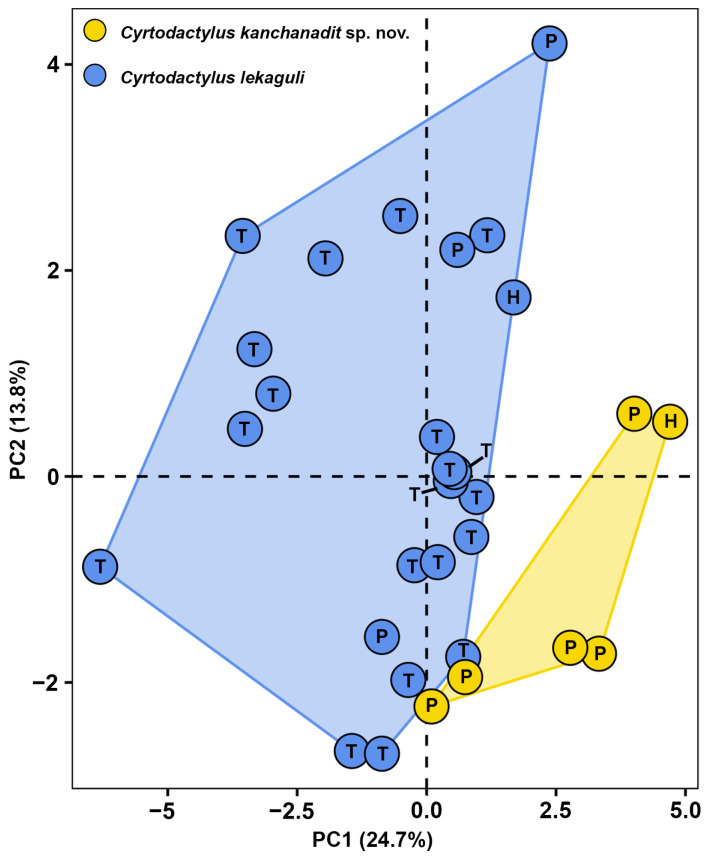
PCA of *Cyrtodactylus kanchanadit* **sp. nov.** and *C. lekaguli* from their type localities. In the scatter plots, the letters signify different specimen types: holotype (H), paratype (P), and topotype (T).

The PCA of two closely related lineages, the Kanchanadit population and *C. lekaguli*, is shown in Figure 3. The Kanchanadit population and *C. lekaguli* cluster separately in the plot of PC1 and PC2, which together explain the largest part of the variance. PC1–PC3 accounted for 48.68% of the morphological variation (Table 2). The factor loadings of PC1 accounted for 24.71%, and were heavily loaded on six characters (TW_adj_, HW_adj_, ES_adj_, EN_adj_, IN_adj_, and PVT). The PC2 accounted for 13.85% of the variation and loaded heavily for AG_adj_ and HD_adj_. The PC3 accounted for 10.13% of the variation and loaded heavily for HL_adj_ and ED_adj_. The PERMANOVA analysis indicated that the Kanchanadit population differed significantly in morphospace from *C. lekaguli* (F = 8.33; R^2^ = 0.229; *p* < 0.001).

**Figure 4:**
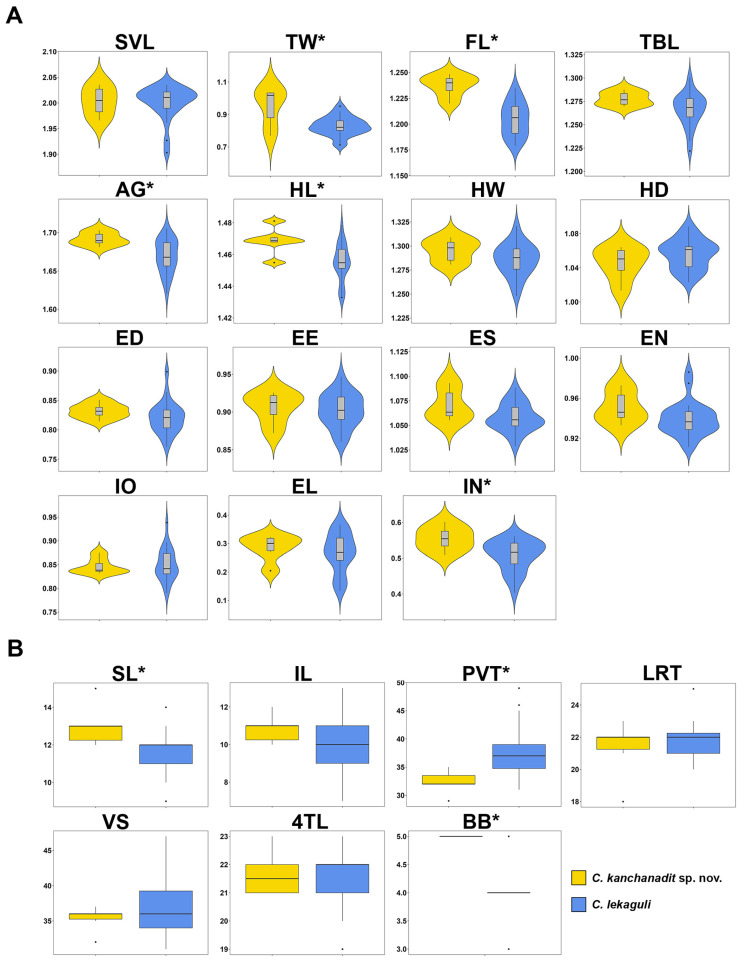
Comparisons of (**A**) 15 mensurements and (**B**) seven meristic characters for *Cyrtodactylus kanchanadit* **sp. nov.** and *C. lekaguli* are summarized in box plots. * denotes significant difference level at *p*-value < 0.05.

The *t*-test (or Mann–Whitney U test) revealed significant differences between the Kanchanadit population and *C. lekaguli* in TW_adj_, FL_adj_, AG_adj_, HL_adj_, IN_adj_, SL, PVT, and BB (*p* < 0.0001–0.0459; Table 3). The Kanchanadit population had larger TW_adj_, FL_adj_, AG_adj_, HL_adj_, and IN_adj_ and higher numbers in SL and BB but lower in PVT than *C. lekaguli*. Mean, standard deviation (SD), and range of size-adjusted characters and the differences between species are summarized (Table 3; Figure 4).

### 3.3. Systematic Account

Based on the evidence of their phylogenetic relationships, relatively high genetic divergence, morphological differences, and diagnostic characters, the Kanchanadit population is hypothesized to be distinct from all congeners in the *C. pulchellus* group and is described below as a new species.

*Cyrtodactylus kanchanadit* sp. nov.

**Figure 5:**
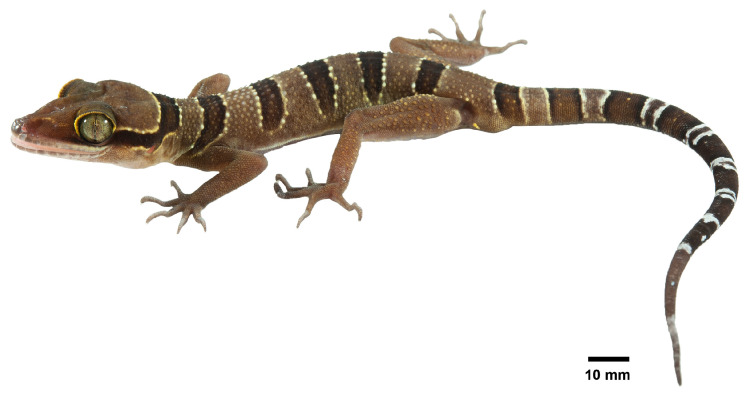
Live specimen of the adult male holotype of *Cyrtodactylus kanchanadit* **sp. nov.** (ZMKU R 01091) from the type locality in Pa Ron Subdistrict, Kanchanadit District, Surat Thani Province, Thailand.

**Figure 6:**
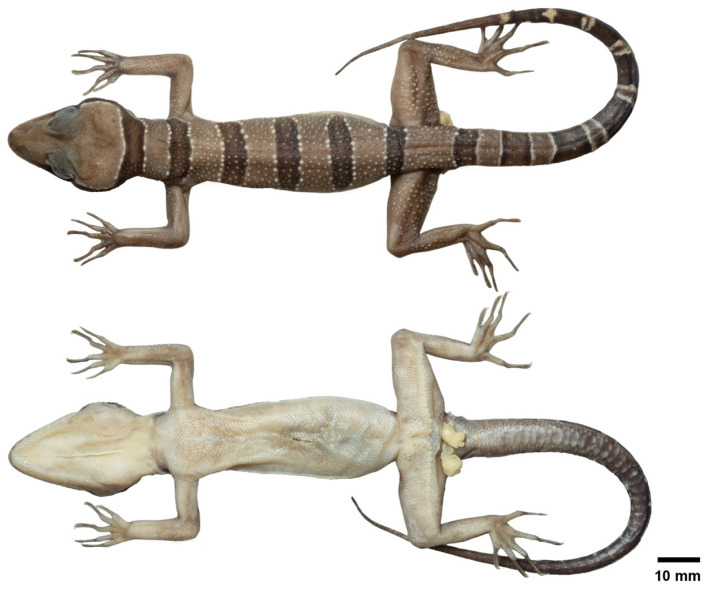
Preserved specimen of the adult male holotype of *Cyrtodactylus kanchanadit* **sp. nov.** (ZMKU R 01091) showing dorsal and ventral views of the specimen.

**Figure 7:**
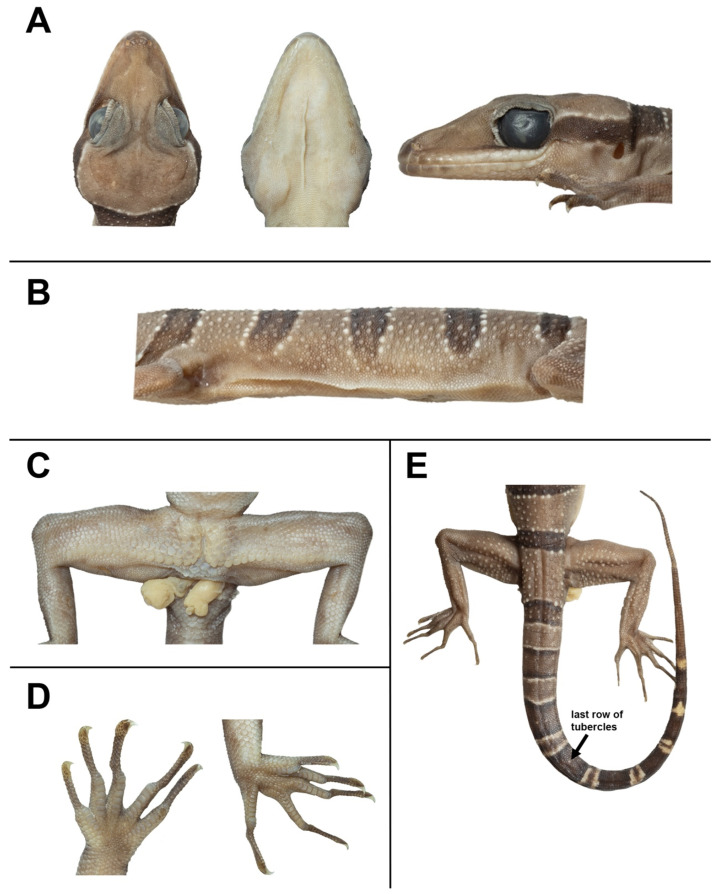
The adult male holotype of *Cyrtodactylus kanchanadit* **sp. nov.** (ZMKU R 01091) in a preservative. (**A**) Head showing dorsal, ventral, and lateral views; (**B**) ventrolateral fold on the left side of the body; (**C**) a depression in the shape of an inverted T in the precloacal region and pore-bearing femoroprecloacal scales; (**D**) ventral views of left manus and left pes; and (**E**) dorsal view of tail showing last low of tubercles.

**Figure 8:**
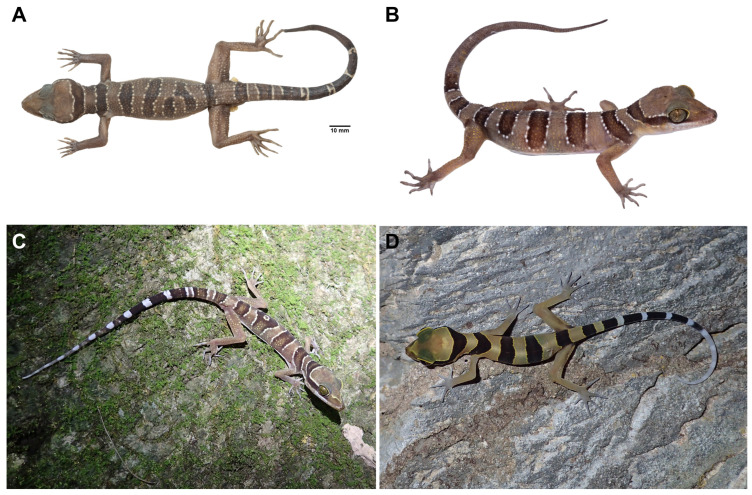
Specimens of *Cyrtodactylus kanchanadit* **sp. nov.** from the type locality show variation in coloration and body pattern. (**A**) Adult male specimen (ZMKU R 01092) in preservative, (**B**) live adult female specimen (ZMKU R 01094), (**C**) an individual from its habitat (not collected) showing four dark dorsal bands with an irregular pattern on the 3rd band, and (**D**) a juvenile (not collected) bearing four dark body bands and having light yellow color on the body with a white tail tip.

Figure 5, Figure 6, Figure 7 and Figure 8

Holotype: Adult male (ZMKU R 01091) collected from Thailand, Surat Thani Province, Kanchanadit District, Pa Ron Subdistrict, Tai Rom Yen National Park, Phet Phanomwat Waterfall (8.94243° N, 99.52816° E; 165 m a.s.l.), on 26 January 2019 by Korkhwan Termprayoon, Anchalee Aowphol, Attapol Rujirawan, Siriporn Yodthong, and Natee Ampai.

Paratypes: Three adult males (ZMKU R 01092–01093 and ZMKU R 01096) and two adult females (ZMKU R 01094–01095); same data as the holotype.

Referred specimens: ZMKU R 01097–01098 (two immature) and ZMKU R 01099 (one juvenile); same data as the holotype.

Etymology: The specific epithet kanchanadit refers to the type locality in Kanchanadit District, Surat Thani Province, Thailand.

Suggested common name: Kanchanadit Bent-toed Gecko.

#### 3.3.1. Diagnosis

*Cyrtodactylus kanchanadit* sp. nov. can be distinguished from all other species of the *C. pulchellus* group by a combination of the following characters: (1) SVL 92.5–101.1 mm in adult males (*n* = 4), 108.2–108.5 mm in adult females (*n* = 2); (2) 11–15 supralabial and 10–12 infralabial scales; (3) weak tuberculation on body; (4) no tubercles on ventral surfaces of forelimbs, gular region, or in ventrolateral body folds; (5) 29–35 paravertebral tubercles; (6) 18–23 longitudinal rows of dorsal tubercles; (7) 32–37 rows of ventral scales; (8) 20–23 subdigital lamellae on the fourth toe; (9) 32–37 femoroprecloacal pores in adult males; (10) absence of precloacal pores in adult females; (11) deep precloacal groove in males; (12) absence of scattered pattern of white tubercles on dorsum; (13) four or five dark dorsal body bands; (14) light caudal bands in adults infused with dark pigmentation; and (15) posterior portion of tail in hatchlings and juveniles white.

#### 3.3.2. Description of Holotype

Adult male 101.1 mm SVL, head moderate in length (HL/SVL 0.29) and wide (HW/HL 0.69), flattened (HD/HL 0.39), distinct from neck, and triangular in dorsal profile; lores concave anteriorly, inflated posteriorly; frontal and prefrontal regions concave; canthus rostralis rounded anteriorly; snout elongate (ES/HL 0.42), rounded in dorsal profile, laterally constricted; eye large (ED/HL 0.23); ear opening oval, obliquely oriented, moderate in size (EL/HL 0.07); eye to ear distance greater than diameter of eye (EE/ED 1.21); rostral rectangular, divided dorsally by an inverted Y-shaped furrow, bordered posteriorly by left and right supranasals and two internasals, bordered laterally by first supralabials; external nares bordered anteriorly by rostral, dorsally by a large anterior supranasal, posteriorly by two moderate postnasals, ventrally by first supralabials; 11/10 (left/right) rectangular supralabials extending to below midpoint of eye, 15/14 to below the posterior margin of the eyeball, decreasing abruptly just posterior to midpoint of eye; 9/8 infralabials extending to below midpoint of eye, 10/12 to upturn the labial margin, decreasing gradually in size posteriorly, usually wider than supralabials; scales of rostrum and lores slightly raised, larger than granular scales on top of head and occiput, and those on the posterior portion of canthus rostralis are slightly larger; scales on top of head and occiput intermixed with rounded, small tubercles extending eyelids; dorsal superciliaries elongate, smooth, largest anteriorly; mental triangular, 3.0 mm in width, 3.7 mm in length, bordered laterally by first infralabials and posteriorly by an enlarged pair of first postmental scales which contact medially for approximately 60% of their length; the second postmental pair smaller than those of the first pair, not in contact with infralabial; one row of slightly enlarged, elongate sublabials extending posteriorly to the seventh (left/right) infralabials; and small, granular, gular scales grading posteriorly into larger, flat, smooth, imbricate, pectoral and ventral scales.

Body relatively short (AG/SVL 0.48) with well-defined, non-tuberculate ventrolateral folds; dorsal scales small, granular, interspersed with low, regularly arranged, weakly keeled tubercles, smaller intervening tubercles rarely present; tubercles extend from the top of head to base of tail extending less than 1/2 of tail; tubercles on the top of head and nape relatively small, and largest on body; 22 longitudinal rows of tubercles at midbody; 35 paravertebral tubercles; 35 flat imbricate ventral scales between ventrolateral body folds; ventral scales larger than dorsal scales; precloacal scales large, smooth; and a deep precloacal groove.

Forelimbs moderately slender, relatively short (FL/SVL 0.16); dorsal scales on forelimbs raised, granular, larger than those on body; dorsal scales on forearm intermixed with enlarged, subconical and weakly keeled tubercles; scales of ventral surface of forearm flat, subimbricate, tubercles absent; palmar scales small, weakly rounded; digits well-developed, inflected at basal, interphalangeal joints; 19/19 (left/right) subdigital lamellae on the fourth finger, 6/6 proximal subdigital lamellae rectangular, broadly expanded proximal to joint inflection, 13/13 distal subdigital lamellae slightly expanded distal to inflection becoming gradually more expanded near the claw; well-developed claws, sheathed by a dorsal and ventral scale; hind limbs more robust than forelimbs, moderate in length (TBL/SVL 0.19), enlarged, subconical, weakly keeled tubercles on dorsal surface of hind limbs separated by smaller juxtaposed scales; ventral scales of thigh flat, smooth, imbricate, larger than dorsal granular scales; ventral, tibial scales flat, smooth, imbricate; a single row of 39 enlarged femoroprecloacal scales extend nearly from knee to knee through precloacal region where they are continuous with enlarged, pore-bearing precloacal scales; 37 continuous pore-bearing femoroprecloacal scales, forming an inverted T bearing a deep, precloacal groove (Figure 7C); eight pore-bearing scales bordering groove (four on each side); postfemoral scales immediately posterior to enlarged scale row small, nearly granular, forming an abrupt union with postfemoral scales on posteroventral margin of thigh; plantar scales weakly rounded to flat; 22/22 (left/right) subdigital lamellae on fourth toe, 8/8 proximal subdigital lamellae rectangular, broadly expanded proximal to joint inflection, 14/14 distal subdigital lamellae slightly expanded distal to inflection becoming gradually more expanded near the claw; and well-developed claws, sheathed by a dorsal and ventral scale.

Regenerated tail 135.8 mm in length, slightly longer than SVL (TL/SVL = 1.34), 10.8 mm in width at base, tapering to a point; dorsal scales of tail flat, squarish; original portion segmented, approximately 8–9 transverse scales rows per segment; one transverse row of two to four dorsal tubercles on the posterior margin of a segment, and four tubercles on the first two segments; caudal tubercles extended to 10th segment of the anterior portion of the original tail (Figure 7E); subcaudal region bearing a large median row of transverse scales; shallow dorsal and lateral caudal furrow; base of tail bearing hemipenial swellings; one row of 3L/3R medium-sized postcloacal tubercles on each hemipenial swelling; and postcloacal scales that are smooth, flat, large, imbricate.

#### 3.3.3. Color of Holotype in Life

Ground color of head, body, and limbs is light brown; superciliaries are light yellow; supralabial and infralabial scales are off-white and gradually dusted with brown anterior to posterior; a wide, dark-brown nuchal band edged anteriorly and posteriorly by thin, yellowish lines bearing scattered creamy-white tubercles extends from the posterior margin of one eye to the posterior margin of another eye; five similar dark-brown body bands between nuchal loop and hind limb insertions edged anteriorly and posteriorly by pale, thin, creamy-white to yellow lines bearing irregular tubercles, and the first band terminates anteriorly to the shoulders, the second to fourth bands terminate just dorsal to the ventrolateral folds, and the fifth band terminates at the groin; dark body bands are thinner than light-colored interspaces; one additional dark-brown band is present posterior to the hind limbs (postsacral band); tubercles on dorsum is similar to body color, and those on the hind limbs are golden brown to light yellow; the ventral surfaces of the head are grayish white, the ground color of the abdomen is white and dusted with brown, and that on limbs is darker; postcloacal tubercles are light yellow; the original portion of the tail bearing seven dark bands is separated by maculate light to dark brown (anteriorly) to white (posteriorly) bands and white caudal band infused with dark pigmentation; the regenerated portion of the tail is light brown with a white marking near the tail tip; subcaudal region is darker than the tail dorsum (Figure 5).

#### 3.3.4. Color of Holotype in Preservative

The overall color pattern of the head, body, limbs, and tail is similar to that in - life, with some fading. The ground color of the head, body, limbs, and dorsum is tan; the dark bands on the dorsum and tail are brown; the yellow-colored tuberculation on dorsum and postcloacal tubercles fades to off-white or white; light-beige colored and dusted with brown on the ventral surface (Figure 6 and Figure 7).

#### 3.3.5. Morphological Variation

The paratypes and referred specimens of *Cyrtodactylus kanchanadit* sp. nov. closely resemble the holotype with some variation in coloration and banding pattern. The body bands of all examined specimens are very similar to those of the holotype with slight differences in shape and size. The fourth dorsal band of ZMKU R 01092 bifurcates on the midline to the left ventrolateral fold (Figure 8A). All specimens have one internasal scale except the holotype possesses two internasal scales. A juvenile (ZMKU R 01099) has a light-yellow ground color of the body with less prominent tubercles, body bands edged by tiny yellowish lines bearing few tubercles, approximately eleven immaculate dark caudal bands on the original tail, and the posterior portion is white. The mensural and meristic data of examined specimens are in Table 4 and Table 5.

In addition, two individuals (not collected) of *Cyrtodactylus kanchanadit* sp. nov. from the type locality were distinct from the holotype in their body pattern. In Figure 8C, an individual has four body bands with an irregular pattern on the third band and shows the original tail with 11 dark caudal bands and 12 white caudal bands. The juvenile (Figure 8D) has four body bands instead of five in the type series.

#### 3.3.6. Distribution and Natural History

**Figure 9:**
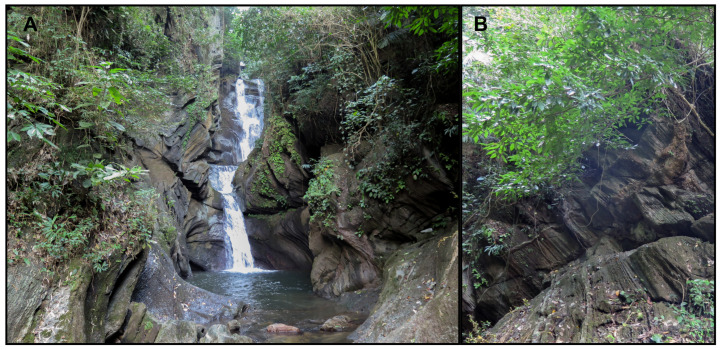
Habitat of *Cyrtodactylus kanchanadit* **sp. nov.** at the type locality Phet Phanomwat Waterfall, Kanchanadit District, Surat Thani Province. (**A**) Waterfall and (**B**) outcrop along the stream bank**.** Photographs by Siriporn Yodthong.

*Cyrtodactylus kanchanadit* **sp. nov.** is currently known from its type locality at Phet Phanomwat Waterfall, Tai Rom Yen National Park, Pa Ron Subdistrict, Kanchanadit District, Surat Thani Province, Thailand (Figure 1 and Figure 9). This type locality is covered by argillaceous limestone [43] and surrounded by lowland dry evergreen forests with large trees, numerous rocks, and boulders along the rocky stream and waterfalls (Figure 9). All geckos were captured from the type locality on 26 January 2019 between 1900 and 2100 h with a temperature of 22.8 °C and relative humidity of 82.9%. Most individuals used rock substrates located in the riparian habitat, except a specimen of ZMKU R 01098, which was found on a midstream rock. Specimen (ZMKU R 01094) is gravid with two eggs (externally visible). This new species is sympatric with other lizard species, including *Cyrtodactylus zebraicus* (Taylor, 1962), *Cnemaspis kamolnorranathi* Grismer, Sumontha, Cota, Grismer, Wood, Pauwels, and Kunya, 2010, *Sphenomorphus maculatus* (Blyth, 1853), *Eutropis macularia* (Blyth, 1853), and *Acanthosaura meridiona* Trivalairat, Sumontha, Kunya, and Chinangkul, 2022.

#### 3.3.7. Comparisons

The distinguishing characters between *Cyrtodactylus kanchanadit* **sp. nov.** and other members in the *C. pulchellus* group are summarized in Table S2. *Cyrtodactylus kanchanadit* **sp. nov.** is differentiated from all seven recognized species of Clade A as it has a combination of diagnostic characteristics, statistical analyses, phylogenetic placement, and genetic divergence in the mitochondrial *ND2* gene.

*Cyrtodactylus kanchanadit* **sp. nov.** can be differentiated from *C. lekaguli* by its statistically significant different values of mensural and meristic characters of TW_adj_, FL_adj_, AG_adj_, HL_adj_, IN_adj_, SL, PVT, and BB (Table 3) and genetic divergence of 3.97–5.55% (Table 1).

*Cyrtodactylus kanchanadit* **sp. nov.** differs from *C. astrum* as it has 7–9 infralabial scales at the middle of the eyeball (vs. 5 or 6 scales); 29–35 paravertebral tubercles between limb insertions (vs. 38–57 tubercles); 11 dark caudal bands (13 or 14 bands); and the absence of a scattered pattern of white tubercles on dorsum (vs. present) and genetic divergence of 8.59–10.93%.

*Cyrtodactylus kanchanadit* **sp. nov.** differs from *C. dayangbuntingensis* as it has a larger maximum SVL of 108.5 mm (vs. 99.0 mm); 32–37 femoroprecloacal pores in males (vs. 26–29 pores); dorsal band interspace ratio of 0.89–1.66 (vs. 0.75); the absence of scattered pattern of white tubercles on dorsum (vs. present); and genetic divergence of 7.99–9.11%.

*Cyrtodactylus kanchanadit* **sp. nov.** differs from *C. langkawiensis* as it has a larger maximum SVL of 108.5 mm (vs. 99.8 mm); 32–37 longitudinal rows of ventral scales (vs. 38–43 scales); 32–37 femoroprecloacal pores in adult males (vs. 30 pores); and genetic divergence of 8.28–10.06%.

*Cyrtodactylus kanchanadit* **sp. nov.** differs from *C. stellatus* as it has a larger maximum SVL of 108.5 mm (vs. 96.1 mm); 32–37 femoroprecloacal pores in males (vs. 24–29 pores); absent of precloacal pores in females (vs. 11–15 pores) and the absence of scattered pattern of white tubercles on dorsum (vs. present); and genetic divergence of 8.09–9.89%.

*Cyrtodactylus kanchanadit* **sp. nov.** differs from *C. sungaiupe* as it has 7 or 8 pore-bearing scales on the preacloacal groove (vs. 5 or 6 pore-bearing scales) and genetic divergence of 8.58–9.84%.

*Cyrtodactylus kanchanadit* **sp. nov.** differs from *C. wangkhramensis* as it has a larger maximum SVL of 108.5 mm (vs. 98.8 mm); 12 light caudal bands (vs. 10 or 11 bands); and genetic divergence of 8.73–10.18%.

## 4. Discussion

In this study, we describe a new species, *Cyrtodactylus kanchanadit* **sp. nov.**, from Southern Thailand based on a combination of molecular genetics, morphological comparisons, and geographic range. The phylogenetic position places this new species as a member of Clade A in the *C. pulchellus* group, and it is closely related to *C. lekaguli*. The genetic divergences between each lineage are relatively high. Furthermore, the species can be distinguished by their distribution across various geographic regions. Most of the species in Clade A are primarily found in karst and cave habitats, with the exception of *C. lekaguli* occurring in forest regions with rocky streams [15,18,20,21]. Morphologically, *Cyrtodactylus kanchanadit* **sp. nov.** closely resembles *C. lekaguli* in body and color pattern. The genetic divergence between *C. lekaguli* and *C. kanchanadit* **sp. nov.** is the smallest of all members of the *C. pulchellus* species group, and they are sister taxa, so it seems most likely that their similarities are inherited from their common ancestor, while the differences are due to some local adaptations. The discovery of *Cyrtodactylus kanchanadit* **sp. nov.** highlights the significant diversity of this well-adapted *Cyrtodactylus* gecko. This brings the *C. pulchellus* group to 20 species and increases the total number of *Cyrtodactylus* species in Thailand to 57 [8,9,10,11,12]. However, populations of the *C. pulchellus* group in some areas of Thailand remain unexplored, including those of *C. lekaguli*. To further understand the taxonomy, species boundary, ecology, and geographic distribution of the *C. pulchellus* group in Thailand, multiple lines of evidence should be applied, and additional surveys in unexplored areas should be conducted.

Grismer [22] noted that color pattern differences among the various populations of *Cyrotdactylus pulchellus* in Peninsular Malaysia (prior to its partitioning into multiple species) likely indicated that it comprised more than one species. It currently contains 20 nominal species (Figure 2) with more species still to be described (Termprayoon et al. in progress). This is similar to the partitioning of *Cyrtodactylus intermedius* into 14 species [10] with more being currently described (Grismer et al. in progress), and *C. irregularis* being partitioned into approximately 35 species [44]. What makes the *pulchellus* group so remarkable is that this high diversity is confined to the narrow-circumscribed Thai–Malay Peninsula from Phuket Island, Thailand, in the north (Figure 2) to Edau-Rompin, Johor, Peninsular Malaysia, in the south [22].

## 5. Conclusions

We described a new species of bent-toed gecko genus *Cyrtodactylus* from Surat Thani Province in Southern Thailand. *Cyrtodactylus kanchanadit* **sp. nov.** is the 20th member of the *Cyrtodactylus pulchellus* group and can be differentiated from its congeners based on its phylogenetic placement, genetic divergences, morphological comparisons, and diagnostic characters. This finding suggests that undiscovered populations may still exist throughout the range. Additional field surveys are needed to understand its species boundary, geographic distribution, and ecology.

## Figures and Tables

**Table 1:** Uncorrected pairwise sequence divergences (*p*-distances) of the Clade A members in the *C. pulchellus* group. *Cyrtodactylus kanchanadit* **sp. nov.** and other species of Clade A in the *C. pulchellus* group based on mitochondrial *ND2* gene and flanking tRNAs. The *p*-distance values are presented as average values with their ranges in parentheses. Distances within the same species are highlighted in bold.

Species	*n*	*Cyrtodactylus kanchanadit* sp. nov.	*C. astrum*	*C. dayangbuntingensis*	*C. langkawiensis*	*C. lekaguli*	*C. stellatus*	*C. sungaiupe*	*C. wangkhramensis*
*Cyrtodactylus* *kanchanadit* **sp. nov**.	4	**1.23** **(0.52–1.78)**							
*C. astrum*	12	9.29 (8.59–10.93)	**1.43** **(0.00–3.10)**						
*C. dayangbuntingensis*	3	8.56 (7.99–9.11)	9.65 (9.25–10.84)	**0.15** **(0.07–0.22)**					
*C. langkawiensis*	10	8.91 (8.28–10.06)	10.32 (9.68–12.63)	7.71 (7.32–8.88)	**0.67** **(0.00–1.88)**				
*C. lekaguli*	5	4.53 (3.97–5.55)	9.64 (8.99–11.33)	8.65 (8.45–8.98)	9.60 (9.20–10.86)	**0.63** **(0.00–1.35)**			
*C. stellatus*	5	8.80 (8.09–9.89)	10.46 (9.79–12.24)	9.58 (9.30–10.40)	10.67 (10.17–12.49)	9.41 (8.83–10.53)	**0.52** **(0.08–1.12)**		
*C. sungaiupe*	7	9.13 (8.58–9.84)	9.87 (9.31–10.84)	8.29 (8.20–8.35)	9.17 (8.79–10.36)	8.87 (8.68–9.21)	9.97 (9.65–10.55)	**0.02** **(0.00–0.07)**	
*C. wangkhramensis*	7	9.36 (8.73–10.18)	9.82 (9.09–11.07)	7.82 (7.53–8.16)	8.60 (8.19–9.65)	9.08 (8.90–9.36)	9.99 (9.44–11.14)	6.74 (6.55–6.89)	**0.31** **(0.00–0.59)**

**Table 2:** Factor loadings for PC1–PC3 analyzing morphological characters of *C. kanchanadit* **sp. nov.** and its sister *C. lekaguli*. Loadings with high values (below −0.6 and over 0.6) are highlighted in bold.

	PC1	PC2	PC3
SVL_adj_	0.038	−0.163	−0.144
TW_adj_	**0.857**	0.098	−0.076
FL_adj_	0.379	−0.475	0.427
TBL_adj_	0.431	0.056	−0.033
AG_adj_	0.283	**−0.653**	−0.094
HL_adj_	0.389	0.302	**0.604**
HW_adj_	**0.854**	0.173	−0.132
HD_adj_	0.294	**0.602**	−0.556
ED_adj_	−0.109	0.285	**0.659**
EE_adj_	0.576	0.307	−0.366
ES_adj_	**0.689**	0.544	0.214
EN_adj_	**0.639**	0.402	0.254
IO_adj_	−0.295	0.490	0.173
EL_adj_	0.070	−0.376	−0.017
IN_adj_	**0.833**	−0.296	−0.140
SL	0.552	−0.090	0.094
IL	0.576	−0.275	−0.118
PVT	**−0.610**	0.381	−0.137
LRT	0.196	0.129	−0.519
VS	0.145	0.570	0.074
4TL	−0.221	0.322	0.231
BB	0.440	−0.310	0.471
Eigenvalue	5.436	3.046	2.228
Percent of variance	24.707	13.845	10.127
Cumulative proportion	24.707	38.553	48.679

**Table 3:** Mean ± SD (min–max) and statistical comparisons of *Cyrtodactylus kanchanadit* **sp. nov.** and *C. lekaguli* from 14 size-adjusted morphometric and seven meristic characters. Statistical comparisons from *t*-test (*t*) and Mann–Whitney U tests. Key: ^a^ tested by Welch’s F test, ^b^ tested by Mann–Whitney U test, and * significant at *p*-value (≤0.05).

Characters	*Cyrtodactylus kanchanadit* sp. nov.		*C. lekaguli*		*t*	*p*
	*n* = 6		*n* = 24			
SVL_adj_	2.00 ± 0.03	(1.97–2.04)	2.00 ± 0.03	(1.9–2.04)	73.500 ^b^	0.9586
TW_adj_	0.95 ± 0.12	(0.77–1.04)	0.83 ± 0.06	(0.71–0.95)	33.000 ^b^	0.0459 *
FL_adj_	1.24 ± 0.01	(1.22–1.25)	1.20 ± 0.02	(1.18–1.23)	−4.632	<0.0001 *
TBL_adj_	1.28 ± 0.01	(1.27–1.29)	1.27 ± 0.02	(1.22–1.30)	−1.522	0.1393
AG_adj_	1.69 ± 0.01	(1.68–1.70)	1.67 ± 0.02	(1.62–1.71)	−3.944 ^a^	0.0007 *
HL_adj_	1.47 ± 0.01	(1.45–1.48)	1.45 ± 0.01	(1.43–1.48)	−2.656	0.0129 *
HW_adj_	1.30 ± 0.01	(1.28–1.31)	1.29 ± 0.02	(1.25–1.32)	−1.331	0.1939
HD_adj_	1.05 ± 0.02	(1.01–1.06)	1.06 ± 0.02	(1.02–1.09)	1.243	0.2242
ED_adj_	0.83 ± 0.01	(0.81–0.85)	0.82 ± 0.03	(0.77–0.90)	−1.013	0.3195
EE_adj_	0.91 ± 0.02	(0.87–0.93)	0.90 ± −0.02	(0.77–0.90)	−0.380	0.6995
ES_adj_	1.07 ± 0.02	(1.06–1.09)	1.06 ± 0.01	(1.03–1.09)	−1.890	0.0692
EN_adj_	0.95 ± 0.02	(0.93–0.97)	0.94 ± 0.02	(0.91–0.99)	−1.569	0.1280
IO_adj_	0.85 ± 0.02	(0.83–0.87)	0.85 ± 0.03	(0.79–0.94)	0.264	0.7936
EL_adj_	0.29 ± 0.04	(0.2–0.32)	0.27 ± 0.07	(0.13–0.37)	−0.617	0.5422
IN_adj_	0.55 ± 0.03	(0.51–0.60)	0.51 ± 0.04	(0.40–0.56)	29.000 ^b^	0.0275 *
SL	13.00 ± 1.10	(12–15)	11.63 ± 1.13	(9–14)	−2.671	0.0125 *
IL	10.83 ± 0.75	(10–12)	10.13 ± 1.57	(7–13)	−1.065	0.2960
PVT	32.33 ± 2.07	(29–35)	37.38 ± 4.33	(31–49)	127.000 ^b^	0.0045 *
LRT	21.33 ± 1.75	(18–23)	21.92 ± 1.1	(20–25)	79.500 ^b^	0.7018
VS	35.33 ± 1.75	(32–37)	37.08 ± 3.96	(31–47)	1.623 ^a^	0.1210
4TL	21.67 ± 0.82	(21–23)	21.42 ± 1.18	(19–23)	67.000 ^b^	0.8077
BB	5.00 ± 0.00	(5)	4.00 ± 0.29	(3–5)	3.000 ^b^	<0.0001 *

**Table 4:** Descriptive morphological characters of type series of *Cyrtodactylus kanchanadit* **sp. nov.** Key: H = holotype; P = paratype; M = male; F = female; Re = regenerated; / = data unavailable or inapplicable; L = left; R = right.

	ZMKU R 01091	ZMKU R 01092	ZMKU R 01093	ZMKU R 01094	ZMKU R 01095	ZMKU R 01096
Type series	H	P	P	P	P	P
Sex	M	M	M	F	F	M
SVL	101.1	101.1	94.5	108.5	108.2	92.5
Tail condition	Re	Re	Re	Re	Re	Re
TL	135.8	107.2	98.4	112.9	91.9	81.8
TW	10.8	10.7	10.3	10.7	10.6	7.0
FL	16.6	17.0	16.8	18.4	18.0	16.8
TBL	19.4	18.7	18.1	20.0	19.3	18.2
AG	48.9	49.1	46.1	55.3	52.4	43.4
HL	29.5	29.6	28.1	29.9	31.7	27.7
HW	20.4	20.2	18.8	21.1	20.4	17.6
HD	11.6	11.4	10.5	12.2	10.9	10.1
ED	6.7	6.9	6.1	7.2	7.8	6.1
EE	8.2	8.4	8.0	8.6	7.6	7.6
ES	12.4	12.3	11.0	12.0	12.2	10.7
EN	9.4	9.3	8.4	9.0	9.3	8.2
IO	4.6	4.1	3.7	4.6	4.7	4.2
EL	2.1	1.6	2.0	1.8	1.9	2.3
IN	4.0	3.7	3.4	3.9	3.8	2.9
SL	15L/14R	12L/13R	13L/12R	13L/13R	12L/12R	13L/11R
SL-mideye	11L/10R	7L/10R	9L/9R	9L/9R	9L/9R	9L/8R
IL	10L/12R	12L/12R	11L/12R	11L/11R	10L/10R	11L/10R
IL-mideye	9L/8R	8L/9R	7L/8R	8L/8R	7L/7R	8L/8R
PVT	35	32	29	34	32	32
LRT	22	21	22	23	18	22
VS	35	37	36	36	36	32
4FLU	13L/13R	13L/13R	13L/12R	14L/15R	13L/13R	16L/16R
4FLE	6L/6R	6L/6R	6L/6R	6L/6R	6L/6R	6L/6R
4FL	19L/19R	19L/19R	19L/18R	20L/21R	19L/19R	22L/22R
4TLU	14L/14R	14L/14R	14L/14R	15L/15R	13L/14R	16L/16R
4TLE	8L/8L	7L/7R	7L/8R	7L/7R	8L/8R	7L/6R
4TL	22L/22R	21L/21R	21L/22R	22L/22R	21L/22R	23L/22R
FPP in males	37	36	34	/	/	32
No of pore-bearing scales on precloacal groove	8(4L/4R)	8(4L/4R)	7(3L/4R)	/	/	7(4L/3R)
PCT rows	1	1	2	/	/	2
No of PCT per row	3L/3R	3L/2R	(2 + 4)L/1 + 3)R	/	/	(1 + 4)L/1 + 3)R
BB	5	5	5	5	5	5
LCB	/	/	/	/	/	/
DCB	/	/	/	/	/	/
Body band/interspace ratio	0.89	1.14	1.38	1.56	1.35	1.66
Deep precloacal groove in male	Yes	Yes	Yes	/	/	Yes
Femoroprecloacal pores continuous	Yes	Yes	Yes	/	/	Yes
Tuberculation	Weak	Weak	Weak	Weak	Weak	Weak
Tubercles on ventral surface of forelimb	No	No	No	No	No	No
Tubercles in gular region	No	No	No	No	No	No
Ventrolateral fold tuberculate	No	No	No	No	No	No
Dorsum bearing scattered pattern of white tubercles	No	No	No	No	No	No
Adult posterior caudal region white	/	/	/	/	/	/
White caudal bands in adults immaculate	No	No	No	No	No	No
Portion of caudal tubercles on original tail	/	/	/	/	/	/

**Table 5:** Descriptive morphological characters of referred specimens of *Cyrtodactylus kanchanadit* **sp. nov.** Key: RF = referred specimens; IM = immature; J = juveniles; / = data unavailable or inapplicable; L = left; R = right.

	ZMKU R 01097	ZMKU R 01098	ZMKU R 01099
	RF	RF	RF
Age	IM	IM	J
SVL	83.3	82.1	64.0
SL	13L/14R	12L/14R	14L/12R
SL-mideye	9L/10R	8L/10R	11L/9R
IL	10L/11R	12L/11R	10L/10R
IL-mideye	7L/7R	7L/7R	7L/7R
PVT	31	32	32
LRT	22	22	22
VS	37	34	33
4FLU	14L/14R	13L/13R	14L/14R
4FLE	6L/6R	6L/6R	6L/6R
4FL	20L/20R	19L/19R	20L/20R
4TLU	14L/14R	13L/14R	14L/14R
4TLE	7L/8R	7L/7R	7L/7R
4TL	21L/22R	20L/21R	21L/21R
BB	5	5	5
LCB	/	/	/
DCB	/	/	/
Body band/interspace ratio	1.52	1.41	/
Tuberculation	Weak	Weak	Weak
Tubercles on ventral surface of forelimb	No	No	No
Tubercles in gular region	No	No	No
Ventrolateral fold tuberculate	No	No	No
Dorsum bearing scattered pattern of white tubercles	No	No	No
Hatchlings/uveniles with white tail tip	/	/	Yes
Portion of caudal tubercles on original tail	/	/	/

## Data Availability

The original contributions presented in this study are included in the article/Supplementary Material. Further inquiries can be directed to the corresponding author (Anchalee Aowphol).

## Supplementary Materials

The following supporting information can be downloaded at: https://www.mdpi.com/article/10.3390/ani14223226/s1, Table S1: Specimens of outgroup and *Cyrtodactylus* species examined in this study [15,16,17,18,19,20,21,24,45]. The institutional abbreviations of examined specimens follow Sabaj [46]. Table S2: Comparative characters of the *C. kanchanadit* **sp. nov.** and the *C. pulchellus* group. The differences from a new species denoted in gray-shaded cells. Keys: W = weak; P = prominent; / = data unavailable. Figure S1: Bayesian Inference analysis of *Cyrtodactylus kanchanadit* **sp. nov.** and the other members of *C. pulchellus* group based on mitochondrial *ND2* and flanking tRNA of 99 specimens. Support values at nodes are Bayesian posterior probabilities (BPPs).

## Abbreviations

SVL	Snout–vent length, taken from the tip of snout to the vent;
TW	Tail width, taken at the base of the tail immediately posterior to the postcloacal swelling;
TL	Tail length, taken from vent to the tip of the tail, original or regenerated;
FL	Forearm length, taken from the posterior margin of the elbow while flexed 90° to the inflection of the flexed wrist;
TBL	Tibia length, taken from the posterior surface of the knee while flexed 90° to the base of the heel;
AG	Axilla to groin length, taken from the posterior margin of the forelimb at its insertion point on the body to the anterior margin of the hind limb at its insertion point on the body;
HL	Head length, the distance from the posterior margin of the retroarticular process of the lower jaw to the tip of the snout;
HW	Head width, measured at the angle of the jaws;
HD	Head depth, the maximum height of head from the occiput to the throat;
ED	Eye diameter, the greatest horizontal diameter of the eyeball;
EE	Eye to ear distance, measured from the anterior edge of the ear opening to the posterior edge of the eyeball;
ES	Eye to snout distance, measured from anterior most margin of the eyeball to the tip of snout;
EN	Eye to nostril distance, measured from the anterior margin of the eyeball to the posterior margin of the external nares;
IO	Inter orbital distance, measured between the anterior edges of the orbit;
EL	Ear length, the greatest vertical distance of the ear opening;
IN	Internarial distance, measured between the nares across the rostrum.
SL	Supralabial scales, counted from the largest scale immediately posterior to the dorsal inflection of the posterior portion of the upper jaw to the rostral scale;
SL-mideye	The numbers of supralabial scales, counted from the largest scale immediately below the middle of the eyeball to the rostral scales;
IL	Infralabial scales, counted from the largest scale immediately posterior to the dorsal inflection of the posterior portion of the upper jaw to the mental scale;
IL-mideye	The numbers of infralabial scales, counted from the largest scale immediately below the middle of the eyeball to the mental scales;
PVT	The number of paravertebral tubercles between limb insertions, counted in a straight line immediately left or right of the vertebral column;
LRT	The number of longitudinal rows of body tubercles, counted transversely across the center of the dorsum from one ventrolateral fold to the other;
VS	The number of longitudinal rows of ventral scales, counted transversely across the center of the abdomen from one ventrolateral fold to the other;
4FLU	The number of small, unmodified subdigital lamellae distal to the digital inflection on the fourth finger, counted from the digital inflection to the claw;
4FLE	The number of expanded subdigital lamellae proximal to the digital inflection on the fourth finger, counted from the base of the first phalanx where it contacts the body of the hand to the largest scale on the digital inflection;
4FL	The total number of subdigital lamellae beneath the fourth finger (4FLU + 4FLE);
4TLU	The number of small, unmodified subdigital lamellae distal to the digital inflection on the fourth toe, counted from the digital inflection to the claw;
4TLE	The number of expanded subdigital lamellae proximal to the digital inflection on the fourth toe, counted from the base of the first phalanx where it contacts the body of the foot to the largest scale on the digital inflection;
4TL	The total number of subdigital lamellae beneath the fourth toe (4TLU + 4TLE);
FPP	The total number of precloacal and femoral pores in male (i.e., the sum of the number of femoral and precloacal scales bearing pores combined as a single meristic referred to as the femoroprecloacal pores);
PCT	The number of rows and total number of postcloacal (hemipenial) tubercles in adult male;
BB	The number of dark body bands between nuchal loop and hind limb insertions;
LCB	The number of light caudal bands on the original tail;
DCB	The number of dark caudal bands on the original tail.
