# Supplementary material for: A New Species of the Cyrtodactylus pulchellus Group (Squamata: Gekkonidae) from Surat Thani Province, Thailand Underscores This Group’s Remarkable Diversity on the Thai-Malay Peninsula
Source: Animals (Basel). 2024 Nov 11;14(22):3226. doi: 10.3390/ani14223226 (PMC11591034; doi:10.3390/ani14223226)
Supplement: Supplementary file 1 [file animals-14-03226-s001.zip › Figure S1_revised_10112024.pdf]

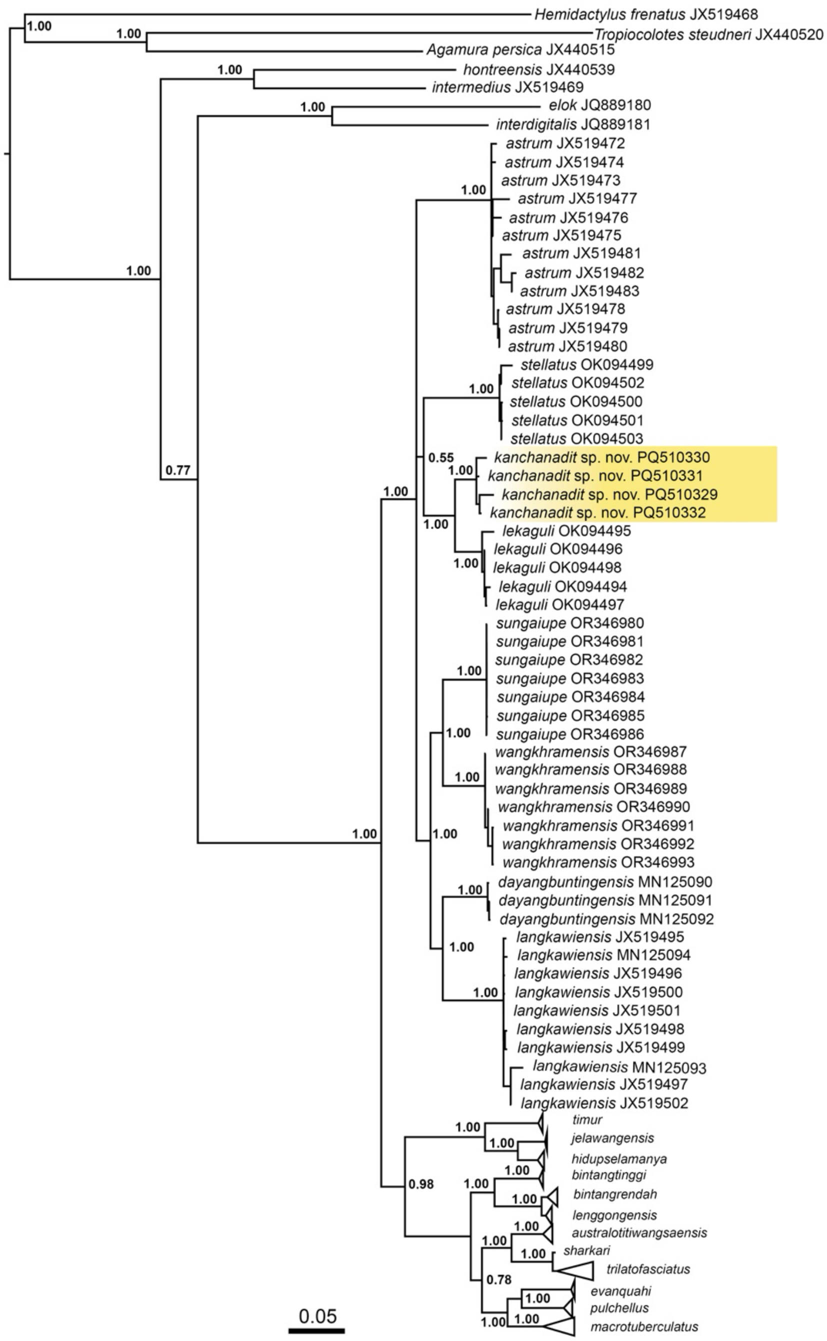

Figure S1. Bayesian Inference analysis of *Cyrtodactylus kanchanadit* **sp. nov.** and the other members of *C. pulchellus* group based on mitochondrial *ND2* and flanking tRNA of 99 specimens. Support values at nodes are Bayesian posterior probabilities (BPPs).
